# Loss of the Metalloprotease ADAM9 Leads to Cone-Rod Dystrophy in Humans and Retinal Degeneration in Mice

**DOI:** 10.1016/j.ajhg.2009.04.005

**Published:** 2009-05-15

**Authors:** David A. Parry, Carmel Toomes, Lina Bida, Michael Danciger, Katherine V. Towns, Martin McKibbin, Samuel G. Jacobson, Clare V. Logan, Manir Ali, Jacquelyn Bond, Rebecca Chance, Steven Swendeman, Lauren L. Daniele, Kelly Springell, Matthew Adams, Colin A. Johnson, Adam P. Booth, Hussain Jafri, Yasmin Rashid, Eyal Banin, Tim M. Strom, Debora B. Farber, Dror Sharon, Carl P. Blobel, Edward N. Pugh, Eric A. Pierce, Chris F. Inglehearn

**Affiliations:** 1 Section of Ophthalmology and Neuroscience, Leeds Institute of Molecular Medicine, St. James's University Hospital, Leeds LS9 7TF, UK; 2 Department of Ophthalmology, Hadassah-Hebrew University Medical Center, 91120 Jerusalem, Israel; 3 Loyola Marymount University, Los Angeles, CA 90045, USA; 4 Eye Department, Chancellor Wing, St James's University Hospital, Leeds LS9 7TF, UK; 5 F.M. Kirby Center for Molecular Ophthalmology, Scheie Eye Institute, University of Pennsylvania School of Medicine, Philadelphia, PA 19104, USA; 6 Hopsital for Special Surgery at Weill Medical College of Cornell University, New York, NY 10021, USA; 7 Peninsula Medical School, Plymouth PL6 8BU, UK; 8 Gene Tech Lab 146/1, Shadman Jail Road, Lahore, 54000, Pakistan; 9 Department of Obstetrics and Gynaecology, King Edward Medical University, Lahore, 54000, Pakistan; 10 Institute of Human Genetics, Helmholtz Zentrum München, German Research Centre for Environmental Health, 85764 Neuherberg, Germany; 11 Jules Stein Eye Institute, UCLA School of Medicine, Los Angeles, CA 90095, USA

## Abstract

Cone-rod dystrophy (CRD) is an inherited progressive retinal dystrophy affecting the function of cone and rod photoreceptors. By autozygosity mapping, we identified null mutations in the ADAM metallopeptidase domain 9 (*ADAM9*) gene in four consanguineous families with recessively inherited early-onset CRD. We also found reduced photoreceptor responses in *Adam9* knockout mice, previously reported to be asymptomatic. In 12-month-old knockout mice, photoreceptors appear normal, but the apical processes of the retinal pigment epithelium (RPE) cells are disorganized and contact between photoreceptor outer segments (POSs) and the RPE apical surface is compromised. In 20-month-old mice, there is clear evidence of progressive retinal degeneration with disorganized POS and thinning of the outer nuclear layer (ONL) in addition to the anomaly at the POS-RPE junction. RPE basal deposits and macrophages were also apparent in older mice. These findings therefore not only identify *ADAM9* as a CRD gene but also identify a form of pathology wherein retinal disease first manifests at the POS-RPE junction.

## Main Text

Cone-rod dystrophy (CRD [MIM #120970]) is a group of genetically and phenotypically heterogenous retinal disorders usually manifesting in childhood or early adulthood. CRD is characterized by predominant or equal loss of cone compared to rod photoreceptors, reduced visual acuity, color-vision abnormalities, photophobia, and visual-field loss. To date, 11 genes and a further six loci have been associated with CRD, which is most commonly inherited in an autosomal-dominant manner (RetNet). So far, only *ABCA4*^1^, ^2^, ^3^ (MIM ^∗^601691) and *RPGRIP1*^4^ (MIM +605446) mutations have been shown to cause nonsyndromic autosomal-recessive CRD, with two other published recessive loci.^5^, ^6^ Only *RPGR* (MIM ^∗^312610) mutations have been associated with X-linked CRD.^7^, ^8^

**Figure 1:**
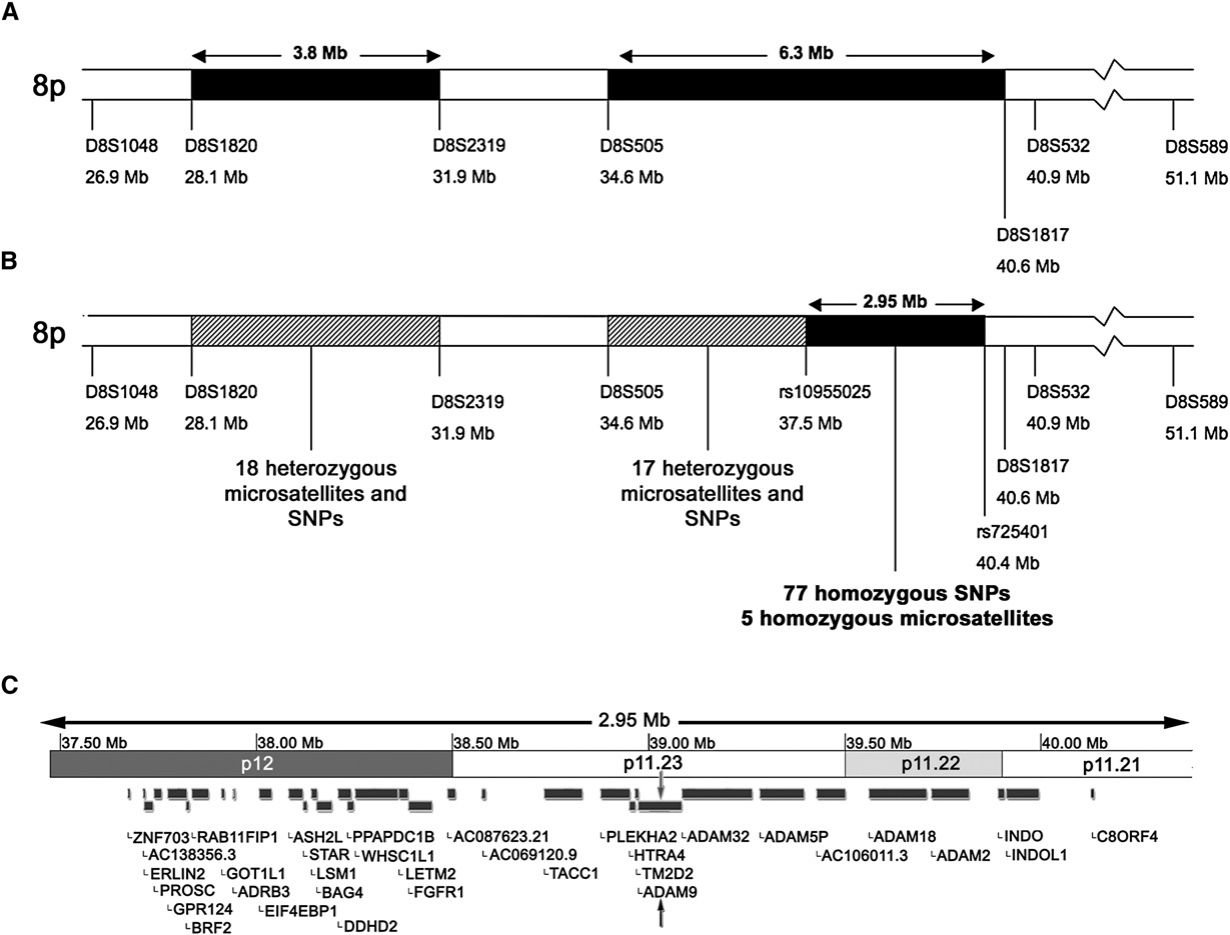
Refinement of the CORD9 Locus (A) Schematic of the CORD9 locus as defined by Danciger et al.^5^ The two published regions of homozygosity are shown shaded in black, with the flanking heterozygous microsatellites marked on either side. (B) Further genotyping of microsatellites and SNPs refined the region so that no block of homozygosity greater than 0.5 Mb remained in the first region and the second region was refined to a 2.95 Mb area of homozygosity at the proximal end of the published interval. (C) A representation of the refined CORD9 region showing 34 potential candidate genes. The following genes were sequenced in this study: *ADAM9* (MIM ^∗^602713), *BAG4* (MIM ^∗^603884), *DDHD2*, *FGFR1* (MIM ^∗^136350), *GPR124* (MIM ^∗^606823), *HTRA4* (MIM ^∗^610700), *PLEKHA2* (MIM ^∗^607773), *RAB11FIP1* (MIM ^∗^608737), *ERLIN2* (MIM ^∗^611605), and *TM2D2* (MIM ^∗^610081).

**Figure 2:**
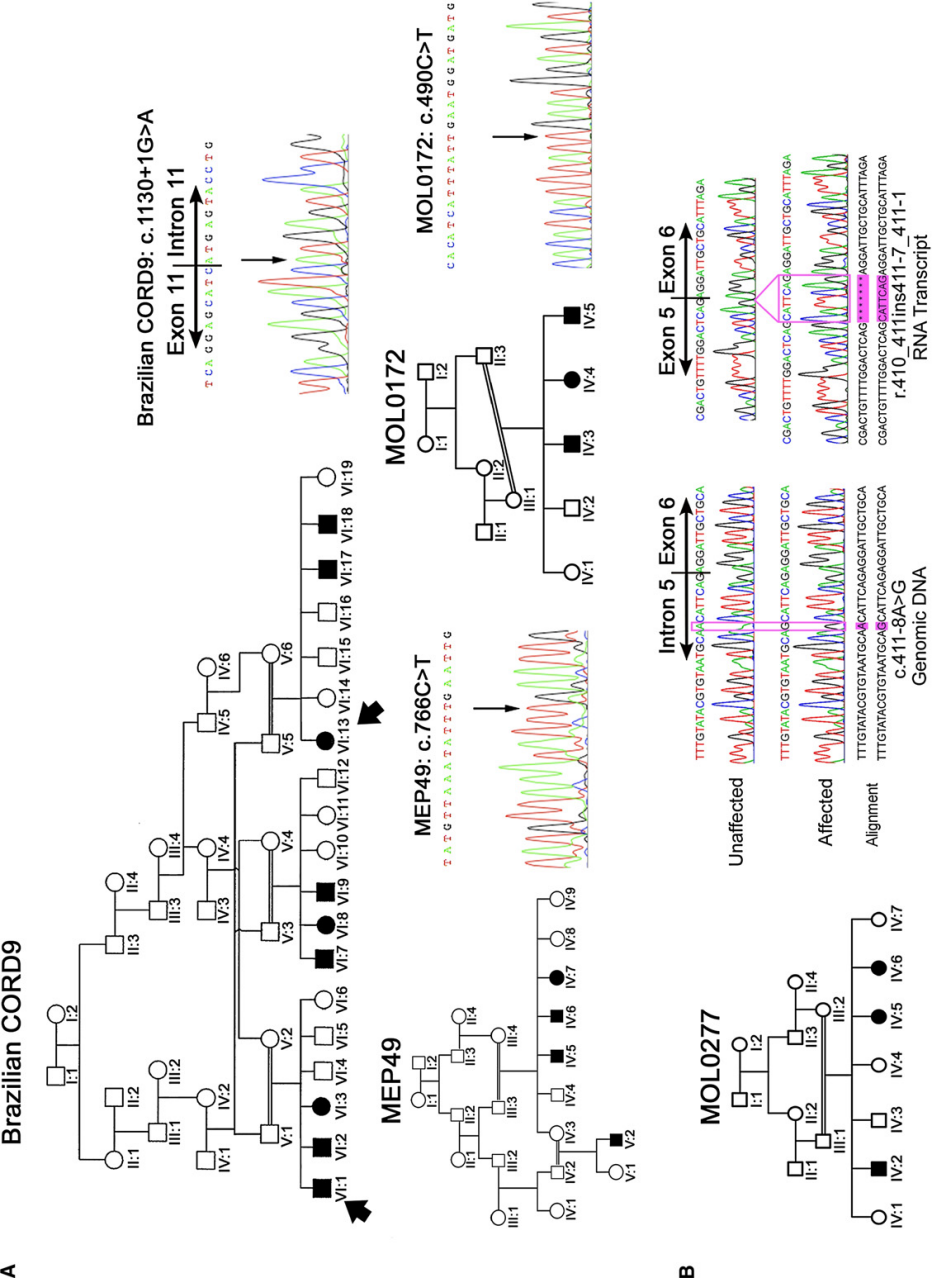
*ADAM9* Mutations in CORD9 Patients (A) Pedigrees of three of the CORD families with genomic DNA sequence of the ADAM9 mutations they carry. The two members of the Brazilian CORD9 family genotyped for refinement of the locus are indicated by arrows. All mutations were shown to segregate with the phenotype in each family by direct sequencing. (B) Pedigree of MOL0277 with genomic and cDNA sequences from mRNA of an affected family member versus an unaffected control individual. cDNA was generated by the Verso cDNA kit according to the manufacturer's protocol. The following primers were designed to amplify through exon 6: forward, GACCTTTTGCCTGAAGATTTTG (5′–3′ located within exon 4); reverse, TCCAAGTAGTTTGCCAGGAG (5′–3′ located within exon 8). The base change at position c.411-8 A→G. and the 7 bp insertion from the 3′ end of intron 5 in the RNA transcript are highlighted.

The CRD locus CORD9 on chromosome 8p11 was first identified in a consanguineous Brazilian family with childhood-onset visual-acuity impairment leading to major loss of central and peripheral vision.^5^ Haplotype analysis revealed a 12 Mb region with two putative homozygous segments (^5^ and Figure 1A). To further refine the locus, we used autozygosity mapping, genotyping over 200 microsatellite markers and single-nucleotide polymorphisms (SNPs) within the published locus in two affected individuals (Table S1, available online). Known microsatellite markers and SNPs were selected from the UCSC genome browser or the International HapMap project. Where possible, SNPs with relatively high levels of heterozygosity were chosen. Microsatellites were genotyped on an ABI PRISM 377 DNA Sequencer and analyzed with Genscan 2.0.2 and Geno Typer 1.1.1 software (Applied Biosciences). SNPs were analyzed by direct sequencing. The individuals genotyped were from different sibships and are indicated by arrows in Figure 2. These data provided support for a 2.95 Mb homozygous segment between rs10955025 and rs725401 containing 34 genes (Figures 1B and 1C).

Candidate genes were chosen on the basis of known expression in the vertebrate retina or eye, homology or functional similarity to known retinal degeneration genes, published studies indicating potential retinal function, and published interactions with proteins thought to be important for retinal function. We amplified genomic DNA by PCR and sequenced ten genes within this region in affected individuals (Figure 1). Sequencing revealed a homozygous point mutation in the *ADAM9* gene (MIM ^∗^602713), altering the first base of intron 11 (c.1130+1G→A) and abolishing the splice-site (Figure 2A). This mutation was excluded in 190 ethnically matched control individuals by restriction digest with BspHI.

Further autozygosity mapping with 10k and 250k Affymetrix SNP arrays and microsatellite markers identified three additional CORD9-linked families of Pakistani (MEP49), Tunisian Jewish (MOL0172), and Arab Muslim (MOL0277) origin. Each was consanguineous and showed a recessive inheritance pattern. Affected individuals in these families reported poor visual acuity in the first decade of life, but nystagmus and photophobia were not noted. Outer retinal atrophy was observed in the macula. Most patients had discrete white patches in the posterior pole and around the optic disc with a pigmentary retinopathy, anterior to the equator. The midperipheral retina showed minimal changes on clinical examination of young patients. In older patients, peripheral pigmentary changes could be observed in some cases. As previously published,^5^ electroretinograms (ERGs) showed a similar degree of rod and cone involvement. The study of human subjects was performed after all individuals provided informed consent. Approval was obtained from the institutional review boards of the participating centers.

Direct sequencing of *ADAM9* in MEP49 revealed a homozygous point mutation in exon 9, creating a premature stop codon (c.766C→T; p.R256X) (Figure 2A). A second homozygous stop codon was identified in MOL0172 (c.490C→T; p.R164X) (Figure 2A). In MOL0277, a homozygous intronic change (c.411-8A→G) was the only potentially pathogenic change identified. Sequence of cDNA derived from patients' peripheral blood lymphocyte mRNA demonstrated that this mutation activated a cryptic splice acceptor site giving rise only to an aberrant transcript (Figure 2B). The mutant transcript has seven additional base pairs of sequence added to the beginning of exon 6 and is predicted to introduce a frameshift resulting in premature termination (R137S*fs*X16). Amplicons from individuals heterozygous and homozygous for the c.766C→T; p.Arg256X mutation were shown to have distinct melting curves by high-resolution melting-curve analysis (HRMCA) when compared to DNA without this mutation (not shown). HRMCA of 138 ethnically matched control individuals confirmed that this mutation was not present in unaffected individuals. The c.490C→T; p.R164X and c.411-8A→G mutations were excluded by direct sequencing in 105 and 160 ethnically matched control individuals, respectively.

**Figure 3:**
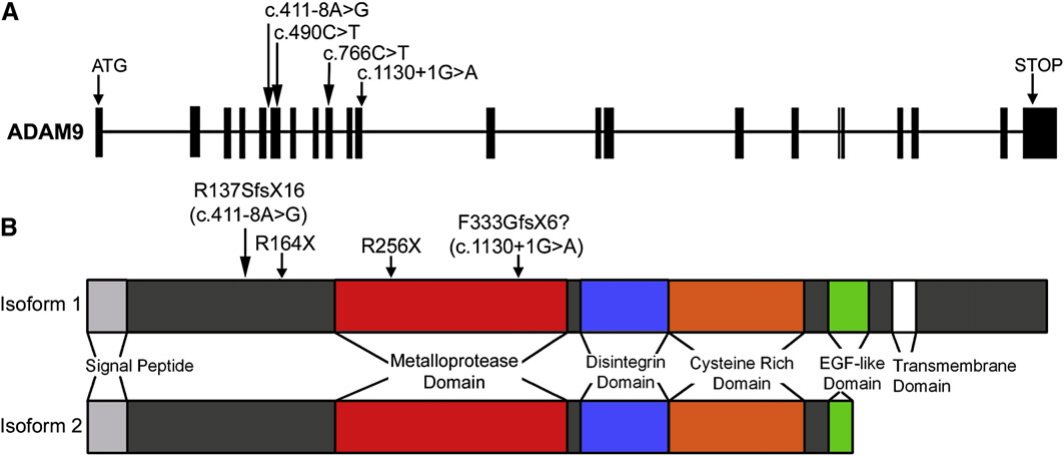
Structure of *ADAM9* and Molecular Consequences of CORD9 Mutations (A) Exon-intron structure of the *ADAM9* gene showing the positions of mutations in CORD9 patients. (B) Domain structure of ADAM9 protein showing the transmembrane and soluble isoforms, with the locations of the CORD9 mutations marked.

**Figure 4:**
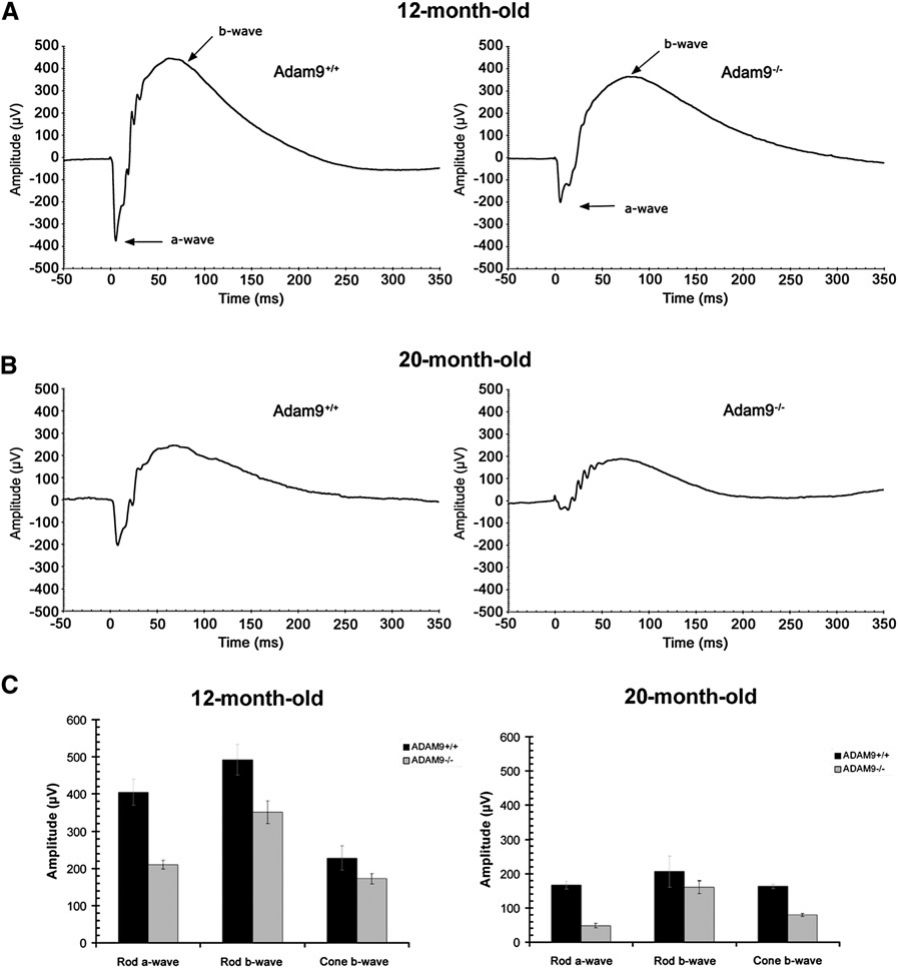
*Adam9*^−/−^ Mice Have Reduced Photoreceptor Responses Compared to Age-Matched Wild-Type Mice Electroretinography was performed as described previously^10^, ^41^ on mice dark adapted for a minimum of 12 hr with pupils dilated with 1% Tropicamide. Full-field ERGs were recorded in a Ganzfeld dome on dark-adapted, anesthetized mice, with caution taken to maintain 37°C body temperature at all times. (A) Representative scotopic ERG traces following ∼1ms light-flash stimulus that isomerized about 1% of the rhodopsin in the rods from 12-month-old mice with stimulus applied at time = 0. (B) Representative scotopic ERG traces from 20-month-old mice with stimulus applied at time = 0. (C) Average scotopic rod a-wave and b-wave amplitudes of 12-month-old and 20-month-old wild-type and *Adam9^−/−^* mice plus photopic cone b-wave amplitudes. Six wild-type and seven *Adam9^−/−^* 12-month-old mice were used in the analysis, and two wild-type and three *Adam9^−/−^* mice were available for the 20 month analysis. Twelve-month-old *Adam9^−/−^* mice had an average a-wave value of approximately 50% compared to age-matched wild-type decreasing to only 30% of age-matched wild-type mice in 20-month-old mice (p < 0.05, Student's t test). Reduction of rod b-wave amplitudes were also significant in 12-month-old knockout mice (p < 0.05, Student's t test), but b-wave data from 20-month-old mice were not statistically significant, probably because of the low number of older mice available to study and the proportionally lesser drop in b-wave response, which was also observed in the 12-month-old mice. Cone b-waves were approximately 75% of wild-type in 12-month-old *Adam9*^−/−^ mice decreasing to 49% in 20-month-old mice, with statistically significant changes (p < 0.05, Student's t test) in the 20-month-old mice. Error bars represent standard error of the mean. See Table 1 for a summary of ERG data.

Two of the human mutations are nonsense changes and the remaining two appear to lead to nonsense changes following splicing defects (Figure 3), strongly suggesting that ADAM9 is likely to be absent in these patients because of nonsense-mediated decay. In order to elucidate the pathogenic mechanism, we therefore investigated previously generated mice that are null for the *Adam9* gene and show no major morphological, histopathological, or behavioral abnormalities during development or early adult life.^9^ We performed ERGs to assess the electrical response of the retina to light in these mice. The rod photoreceptor response of 12-month-old *Adam9^−/−^* mice, as measured by the dark-adapted a-wave,^10^ showed a saturated response of approximately 50% the amplitude of wild-type mice (Figures 4A and 4C, Table 1). Twenty-month-old *Adam9^−/−^* mice had saturated a-wave responses approximately 30% the amplitude of the age-matched wild-type (Figures 4B and 4C, Table 1), suggesting a progressive retinal degeneration. Rod b-waves, reflecting the depolarizing response of rod bipolar cells, were less severely but significantly reduced in *Adam9^−/−^* mice (Figure 4, Table 1). Although reductions of cone-driven b-wave responses were not statistically significant in 12-month-old mice, probably because of the low proportion of cone photoreceptors in the mouse retina, 20-month-old mice had proportionally greater reductions compared to age-matched wild-types, with statistically significant Student's t test p values (Figure 4C, Table 1). These data suggest a progressive degeneration affecting both rods and cones in *Adam9^−/−^* mice.

**Table 1:** Summary of ERG Data

	12 Month			20 Month		
	*Adam9^+/+^*	*Adam9^−/−^*	t Test	*Adam9^+/+^*	*Adam9^−/−^*	t Test
Rod a-wave (μV)	405 ± 35	210 ± 12	0.0059	167 ± 48	48 ± 6	0.0457
Rod b-wave (μV)	492 ± 41	351 ± 31	0.0304	206 ± 46	160 ± 18	0.6067
Cone b-wave (μV)	228 ± 33	172 ± 14	0.2141	163 ± 6	80 ± 4	0.03515

Average amplitudes of rod a-waves, rod b-waves, and cone b-waves ± standard error (SE) are shown for each age group and genotype. Student's t test p values indicate statistical significance.

**Figure 5:**
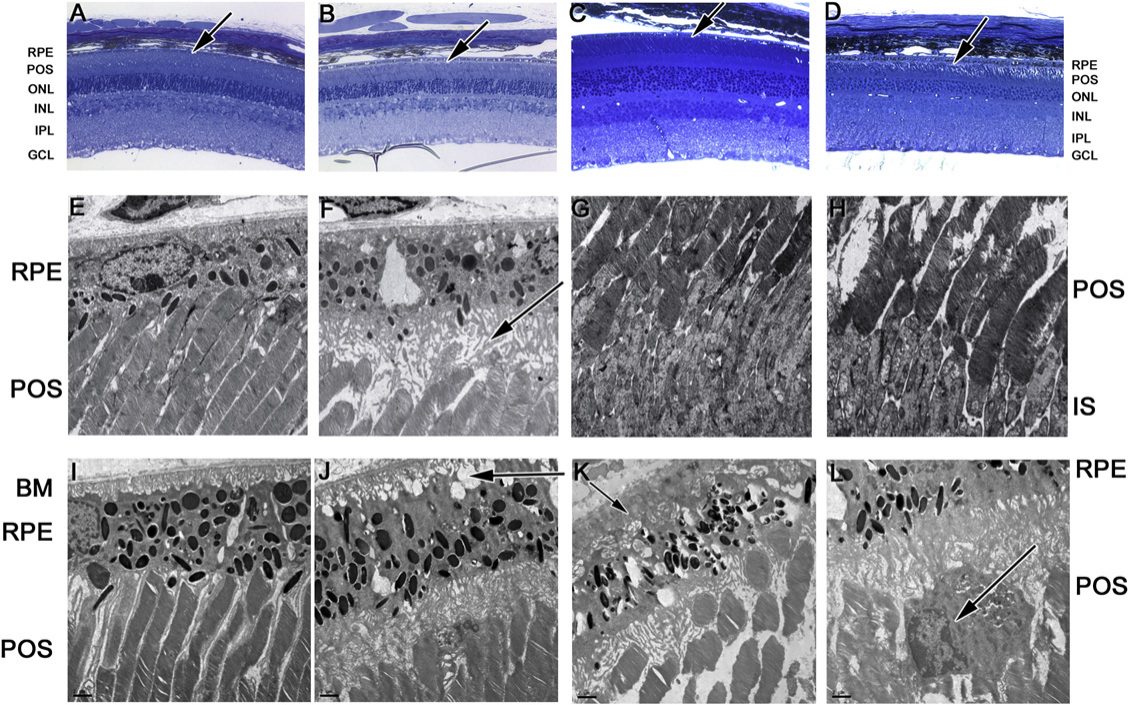
Physical Abnormalities of the *Adam9^−/−^* Retina Eye cups from three knockout and three wild-type mice of each age were fixed in 2% paraformaldehyde + 2% glutaraldehyde in 0.2 M sodium cacodylate buffer (pH 7.4) for 4 hr before being trimmed and postfixed in 1% osmium tetroxide. Tissues were dehydrated in a graded ethanol series, infiltrated, and embedded in Epon resin (EMbed812; Electron Microscopy Sciences) according to the manufacturer's instructions. (A–D) Sections of 1 μm thickness were cut and stained with alkaline toluidine blue for light microscopy. Twelve-month-old wild-type retinas (A) and 20-month-old wild-type retinas (C) show normal retinal structure with a tight interface between the POS and RPE (indicated by arrows). (B) Twelve-month-old *Adam9*^−/−^ mice show otherwise normal retinal histology except for an abnormal gap between the POS and RPE, indicated by an arrow. (D) Twenty-month-old *Adam9^−/−^* mice had the same abnormal interface but also displayed disorganized POS and thinning ONL, indicative of degeneration. (E–L) Ultrathin sections of 60 to 80 nm thickness were cut, stained with lead citrate and uranyl acetate, and examined with a Tecnai transmission electron microscope. Electron microscopy shows (E) normal POS-RPE interfaces in 12-month-old wild-type retinas compared with (F) abnormal interfaces between the POS and RPE (arrow) in 12-month-old *Adam9^−/−^* retinas. Comparison of 12-month-old (G) wild-type and (H) *Adam9^−/−^* POS and IS showed that the photoreceptors appeared structurally normal. (I) Twenty-month-old wild-type retinas show normal morphology. (J) In addition to abnormal POS-RPE interfaces, the basal surface of the RPE shows abnormal infoldings (arrow) in 20-month-old Adam9^−/−^ eyes. (K) In some 20-month-old *Adam9*^−/−^ sections, electron-dense deposits between BM and the RPE were observed (arrow). Disorganized photoreceptor outer segments are visible in addition to the abnormal OS-RPE interface. (L) Tissue macrophages (arrow) were observed in 20-month-old *Adam9^−/−^* retinas. The following abbreviations are used: ONL, outer nuclear layer; POS, photoreceptor outer segments; IS, photoreceptor inner segments; RPE, retinal pigment epithilium; and BM, Bruch's membrane.

Histological analysis of retinas from 12-month-old *Adam9^−/−^* mice showed an abnormal gap between the POS and RPE (Figure 5B). Electron microscopy revealed extended malformed, vesiculated RPE apical processes (Figures 5E and 5F) and disrupted contact with the POS. However, photoreceptors and other neuronal layers appeared structurally normal at this age. Analysis of 20-month-old *Adam9^−/−^* mice provided evidence for further degeneration. In addition to the POS-RPE interface abnormalities observed at 12 months, these mice had disorganized POS and a thinning outer nuclear layer (ONL) (Figure 5D), macrophages within the gap between the POS and the RPE (Figure 5L), and unusual infoldings of the basal membrane of the RPE (Figure 5J). In some slides, material could also be seen deposited between the RPE and Bruch's membrane (Figure 5K). These histological findings reveal a progressive degeneration consistent with ERG analyses and implicate the POS-RPE junction as the site at which the pathology first manifests. A defect in the extracellular matrix (ECM) between the POS and the RPE may be responsible for the initial defects in photoreceptor signaling detected by ERGs, in a similar manner to that which has been suggested by analysis of *Slc16a8^−/−^* mice.^11^ It is intriguing to speculate that the basal deposits observed (Figure 5K) may be analogous to the drusen deposits observed in human retinal degenerations, such as CRD and age-related macular degeneration (AMD). It is also interesting to note that macrophages have been implicated in the pathology of AMD.

We performed immunofluorescence on mouse retinas to establish Adam9 expression in the eye. Although we observed the brightest Adam9 staining at the apical surface of the RPE (Figure S1), the site of observed pathology in mice, staining of some sections from *Adam9^−/−^* mice indicated cross-reactivity at the same site. These data should therefore be interpreted with caution.

ADAM9 is a widely expressed and particularly polyvalent member of the multifunctional ADAM family of proteins. It has been implicated in cell-matrix interactions,^12^ ECM remodeling,^13^ alpha-secretase activity for amyloid precursor protein (APP),^14^ HB-EGF shedding,^15^ and the pathology of cancer^16^, ^17^, ^18^ and Alzheimer disease,^14^ but until now a physiological role remained elusive. It has been suggested previously that absence of an ortholog in *Drosophila melanogaster* and *Caenorhabditis elegans* may indicate that ADAM9 functions in cells or organs that are more highly evolved in vertebrates,^9^ and our data support the idea that ADAM9 fulfills a specific role in the vertebrate retina. Whereas dysregulation of members of the ADAM family has been implicated in the pathogenesis of diseases such as Alzheimer, cancer, rheumatoid arthritis, and asthma (reviewed in ^19^), and *ADAM9* has been shown to be downregulated in cataracts,^20^ to the best of our knowledge no other germinal mutation in an *ADAM* has been shown to be the direct cause of disease.

Our findings could suggest a defect in adhesion at the POS-RPE interface in the absence of Adam9. Indeed, Adam9 has been shown to function as an adhesion molecule by binding to α_v_β_5_ integrin.^21^ α_v_β_5_ integrin, the only integrin localized to the apical surface of the RPE,^22^, ^23^ is required for retinal adhesion^24^ and phagocytosis of POS.^23^ In addition, integrin *β_5_*^−/−^ mice have age-related vision loss.^25^ Adam9 at the POS-RPE junction could therefore interact with α_v_β_5_ integrin and may mediate adhesion of POS to the RPE and efficient uptake of shed outer segments. However, ERGs in the *Adam9*^−/−^ mice appear less severely disturbed than those of *β_5_^−/−^* mice, whereas *β_5_^−/−^* mice lack the morphological abnormalities observed at the RPE-POS junction in *Adam9^−/−^* mice. This suggests that Adam9 has an additional role in the retina independent of α_v_β_5_ integrin, possibly in the remodeling of the ECM between the RPE and POS or in the shedding of factors essential for the maintenance of the ECM.

Interestingly, genes adjacent to *ADAM9* on chromosome 8 (Figure 1C) display a degree of paralogy with the locus on chromosome 10 implicated in AMD,^26^, ^27^ the functional variant of which is still debated.^28^, ^29^, ^30^, ^31^, ^32^ The *TACC2* (MIM ^∗^605302), *PLEKHA1* (MIM ^∗^607772), and *HTRA1* (MIM ^∗^602194) genes at the AMD-associated locus on chromosome 10 have paralogs in the form of the *TACC1* (MIM ^∗^605301), *PLEKHA2* (MIM ^∗^607773), and *HTRA4* (MIM ^∗^610700) genes adjacent to *ADAM9* at the CORD9 locus on chromosome 8. However, there is no *ADAM* family member at the AMD locus on chromosome 10. Nevertheless, the fact that these genes lie in the same orientation relative to each other at each locus and that *ADAM9* is the next-but-one gene in the same orientation raises an intriguing if speculative possibility that a shared regulatory element for these loci may influence the transcription of *ADAM9*^33^.

Several components of the ECM have been implicated in AMD or AMD-related disorders.^31^, ^34^, ^35^, ^36^, ^37^ Mice with a homozygous mutation in one of these, *TIMP3* (MIM ^∗^188826), encoding an inhibitor of metalloproteases, appear to show disturbances of the apical processes of the RPE in addition to basal infoldings, similar to the changes observed in *Adam9^−/−^* mice.^38^ Although ADAM9 is thought not to be inhibited by TIMP3,^39^ our data suggest that it may be worthwhile to re-evaluate the possibility of an interaction between these molecules. Furthermore, drusen deposits from cases of AMD have been shown to contain amyloid beta^40^ and some *Adam9*^−/−^ mice appear to have drusen-like deposits, so it is tempting to consider that loss of ADAM9, a protease with alpha-secretase activity potentially contributing to the nonamyloidogenic pathway of APP processing, could result in an increase in amyloid beta production. Further analysis of basal deposits in the *Adam9*^−/−^ retina will be required to test this theory.

In summary, we have demonstrated a pathology localized to the POS-RPE junction and leading to retinal degeneration in *Adam9*^−/−^ mice and shown that *ADAM9* mutations cause retinal degeneration in human patients. The milder phenotype of *Adam9*^−/−^ mice is likely to represent the early stages of the human disease because of a combination of shorter life span, the progressive nature of CORD, and the fact that mice lack a macula, instead showing uniform photoreceptor density. Indeed, the comparatively mild mouse phenotype suggests that the most important function of ADAM9 may be in areas of high photoreceptor density or associated with cones. Whereas several mouse models have exhibited varying forms of retinal degeneration wherein the photoreceptors are not the initial site of pathology, to the best of our knowledge the *Adam9^−/−^* phenotype is unique and no human disease has been previously linked to such a phenotype. Our data therefore uncover a physiological role for ADAM9, reveal what we believe may be a novel causative pathway for human retinal degeneration, and highlight a potentially overlooked pathological feature of retinal degenerations, including CRD and AMD. Finally, the observation that photoreceptors appear to be intact in the early stages of the disease also suggests that *ADAM9* may be a valid target for gene therapy.

## Web Resources

The URLs for data presented herein are as follows:

International HapMap Project, http://www.hapmap.org/
Online Mendelian Inheritance in Man (OMIM), http://www.ncbi.nlm.nih.gov/Omim/
RetNet, the Retinal Information Network, http://www.sph.uth.tmc.edu/RetNet/
UCSC Genome Browser, http://genome.ucsc.edu/

## Supplemental Data

Document S1. One Table and One Figure

## References

[1] Cremers F.P., van de Pol D.J., van Driel M., den Hollander A.I., van Haren F.J., Knoers N.V., Tijmes N., Bergen A.A., Rohrschneider K., Blankenagel A. (1998). Autosomal recessive retinitis pigmentosa and cone-rod dystrophy caused by splice site mutations in the Stargardt's disease gene ABCR. Hum. Mol. Genet..

[2] Maugeri A., Klevering B.J., Rohrschneider K., Blankenagel A., Brunner H.G., Deutman A.F., Hoyng C.B., Cremers F.P. (2000). Mutations in the ABCA4 (ABCR) gene are the major cause of autosomal recessive cone-rod dystrophy. Am. J. Hum. Genet..

[3] Ducroq D., Rozet J.-M., Gerber S., Perrault I., Barbet F., Hanein S., Hakiki S., Dufier J.-L., Munnich A., Hamel C. (2002). The ABCA4 gene in autosomal recessive cone-rod dystrophies. Am. J. Hum. Genet..

[4] Hameed A., Abid A., Aziz A., Ismail M., Mehdi S.Q., Khaliq S. (2003). Evidence of RPGRIP1 gene mutations associated with recessive cone-rod dystrophy. J. Med. Genet..

[5] Danciger M., Hendrickson J., Lyon J., Toomes C., McHale J.C., Fishman G.A., Inglehearn C.F., Jacobson S.G., Farber D.B. (2001). CORD9 a new locus for arCRD: Mapping to 8p11, estimation of frequency, evaluation of a candidate gene. Invest. Ophthalmol. Vis. Sci..

[6] Khaliq S., Hameed A., Ismail M., Anwar K., Leroy B.P., Mehdi S.Q., Payne A.M., Bhattacharya S.S. (2000). Novel locus for autosomal recessive cone-rod dystrophy CORD8 mapping to chromosome 1q12–Q24. Invest. Ophthalmol. Vis. Sci..

[7] Demirci F.Y.K., Rigatti B.W., Wen G., Radak A.L., Mah T.S., Baic C.L., Traboulsi E.I., Alitalo T., Ramser J., Gorin M.B. (2002). X-linked cone-rod dystrophy (locus COD1): Identification of mutations in RPGR exon ORF15. Am. J. Hum. Genet..

[8] Ebenezer N.D., Michaelides M., Jenkins S.A., Audo I., Webster A.R., Cheetham M.E., Stockman A., Maher E.R., Ainsworth J.R., Yates J.R. (2005). Identification of novel RPGR ORF15 mutations in X-linked progressive cone-rod dystrophy (XLCORD) families. Invest. Ophthalmol. Vis. Sci..

[9] Weskamp G., Cai H., Brodie T.A., Higashyama S., Manova K., Ludwig T., Blobel C.P. (2002). Mice lacking the metalloprotease-disintegrin MDC9 (ADAM9) have no evident major abnormalities during development or adult life. Mol. Cell. Biol..

[10] Lyubarsky A.L., Falsini B., Pennesi M.E., Valentini P., Pugh E.N. (1999). UV- and midwave-sensitive cone-driven retinal responses of the mouse: A possible phenotype for coexpression of cone photopigments. J. Neurosci..

[11] Daniele L.L., Sauer B., Gallagher S.M., Pugh E.N., Philp N.J. (2008). Altered visual function in monocarboxylate transporter 3 (Slc16a8) knockout mice. Am. J. Physiol. Cell Physiol..

[12] Mahimkar R.M., Visaya O., Pollock A.S., Lovett D.H. (2005). The disintegrin domain of ADAM9: A ligand for multiple beta1 renal integrins. Biochem. J..

[13] Schwettmann L., Tschesche H. (2001). Cloning and expression in Pichia pastoris of metalloprotease domain of ADAM 9 catalytically active against fibronectin. Protein Expr. Purif..

[14] Asai M., Hattori C., Szabo B., Sasagawa N., Maruyama K., Tanuma S., Ishiura S. (2003). Putative function of ADAM9, ADAM10, and ADAM17 as APP alpha-secretase. Biochem. Biophys. Res. Commun..

[15] Izumi Y., Hirata M., Hasuwa H., Iwamoto R., Umata T., Miyado K., Tamai Y., Kurisaki T., Sehara-Fujisawa A., Ohno S. (1998). A metalloprotease-disintegrin, MDC9/meltrin-gamma/ADAM9 and PKCdelta are involved in TPA-induced ectodomain shedding of membrane-anchored heparin-binding EGF-like growth factor. EMBO J..

[16] Grutzmann R., Luttges J., Sipos B., Ammerpohl O., Dobrowolski F., Alldinger I., Kersting S., Ockert D., Koch R., Kalthoff H. (2004). ADAM9 expression in pancreatic cancer is associated with tumour type and is a prognostic factor in ductal adenocarcinoma. Br. J. Cancer.

[17] Carl-McGrath S., Lendeckel U., Ebert M., Roessner A., Rocken C. (2005). The disintegrin-metalloproteinases ADAM9, ADAM12, and ADAM15 are upregulated in gastric cancer. Int. J. Oncol..

[18] Peduto L., Reuter V.E., Shaffer D.R., Scher H.I., Blobel C.P. (2005). Critical function for ADAM9 in mouse prostate cancer. Cancer Res..

[19] Edwards D.R., Handsley M.M., Pennington C.J. (2008). The ADAM metalloproteinases. Mol. Aspects Med..

[20] Lim J.M., Lee J.H., Wee W.R., Joo C.K. (2002). Downregulated expression of ADAM9 in anterior polar cataracts. J. Cataract Refract. Surg..

[21] Zhou M., Graham R., Russell G., Croucher P.I. (2001). MDC-9 (ADAM-9/Meltrin [gamma]) functions as an adhesion molecule by binding the [alpha]v[beta]5 integrin. Biochem. Biophys. Res. Commun..

[22] Anderson D.H., Johnson L.V., Hageman G.S. (1995). Vitronectin receptor expression and distribution at the photoreceptor-retinal pigment epithelial interface. J. Comp. Neurol..

[23] Finnemann S.C., Bonilha V.L., Marmorstein A.D., Rodriguez-Boulan E. (1997). Phagocytosis of rod outer segments by retinal pigment epithelial cells requires alpha vbeta 5 integrin for binding but not for internalization. Proc. Natl. Acad. Sci. USA.

[24] Nandrot E.F., Anand M., Sircar M., Finnemann S.C. (2006). Novel role for {alpha}vbeta5-integrin in retinal adhesion and its diurnal peak. Am. J. Physiol. Cell Physiol..

[25] Nandrot E.F., Kim Y., Brodie S.E., Huang X., Sheppard D., Finnemann S.C. (2004). Loss of synchronized retinal phagocytosis and age-related blindness in mice lacking {alpha}v{beta}5 integrin. J. Exp. Med..

[26] Weeks D.E., Conley Y.P., Mah T.S., Paul T.O., Morse L., Ngo-Chang J., Dailey J.P., Ferrell R.E., Gorin M.B. (2000). A full genome scan for age-related maculopathy. Hum. Mol. Genet..

[27] Weeks D.E., Conley Y.P., Tsai H.-J., Mah T.S., Schmidt S., Postel E.A., Agarwal A., Haines J.L., Pericak-Vance M.A., Rosenfeld P.J. (2004). Age-related maculopathy: A genomewide scan with continued evidence of susceptibility loci within the 1q31, 10q26, and 17q25 regions. Am. J. Hum. Genet..

[28] Rivera A., Fisher S.A., Fritsche L.G., Keilhauer C.N., Lichtner P., Meitinger T., Weber B.H.F. (2005). Hypothetical LOC387715 is a second major susceptibility gene for age-related macular degeneration, contributing independently of complement factor H to disease risk. Hum. Mol. Genet..

[29] Jakobsdottir J., Conley Y.P., Weeks D.E., Mah T.S., Ferrell R.E., Gorin M.B. (2005). Susceptibility genes for age-related maculopathy on chromosome 10q26. Am. J. Hum. Genet..

[30] DeWan A., Liu M., Hartman S., Zhang S.S.-M., Liu D.T.L., Zhao C., Tam P.O.S., Chan W.M., Lam D.S.C., Snyder M. (2006). HTRA1 promoter polymorphism in wet age-related macular degeneration. Science.

[31] Yang Z., Camp N.J., Sun H., Tong Z., Gibbs D., Cameron D.J., Chen H., Zhao Y., Pearson E., Li X. (2006). A variant of the HTRA1 gene increases susceptibility to age-related macular degeneration. Science.

[32] Fritsche L.G., Loenhardt T., Janssen A., Fisher S.A., Rivera A., Keilhauer C.N., Weber B.H.F. (2008). Age-related macular degeneration is associated with an unstable ARMS2 (LOC387715) mRNA. Nat. Genet..

[33] Spilianakis C.G., Lalioti M.D., Town T., Lee G.R., Flavell R.A. (2005). Interchromosomal associations between alternatively expressed loci. Nature.

[34] Weber B.H., Vogt G., Pruett R.C., Stohr H., Felbor U. (1994). Mutations in the tissue inhibitor of metalloproteinases-3 (TIMP3) in patients with Sorsby's fundus dystrophy. Nat. Genet..

[35] Stone E.M., Lotery A.J., Munier F.L., Heon E., Piguet B., Guymer R.H., Vandenburgh K., Cousin P., Nishimura D., Swiderski R.E. (1999). A single EFEMP1 mutation associated with both Malattia Leventinese and Doyne honeycomb retinal dystrophy. Nat. Genet..

[36] Schultz D.W., Klein M.L., Humpert A.J., Luzier C.W., Persun V., Schain M., Mahan A., Runckel C., Cassera M., Vittal V. (2003). Analysis of the ARMD1 locus: Evidence that a mutation in HEMICENTIN-1 is associated with age-related macular degeneration in a large family. Hum. Mol. Genet..

[37] Stone E.M., Braun T.A., Russell S.R., Kuehn M.H., Lotery A.J., Moore P.A., Eastman C.G., Casavant T.L., Sheffield V.C. (2004). Missense variations in the fibulin 5 gene and age-related macular degeneration. N. Engl. J. Med..

[38] Weber B.H.F., Lin B., White K., Kohler K., Soboleva G., Herterich S., Seeliger M.W., Jaissle G.B., Grimm C., Reme C. (2002). A mouse model for Sorsby fundus dystrophy. Invest. Ophthalmol. Vis. Sci..

[39] Amour A., Knight C.G., English W.R., Webster A., Slocombe P.M., Knäuper V., Docherty A.J.P., Becherer J.D., Blobel C.P., Murphy G. (2002). The enzymatic activity of ADAM8 and ADAM9 is not regulated by TIMPs. FEBS Lett..

[40] Johnson L.V., Leitner W.P., Rivest A.J., Staples M.K., Radeke M.J., Anderson D.H. (2002). The Alzheimer's A beta -peptide is deposited at sites of complement activation in pathologic deposits associated with aging and age-related macular degeneration. Proc. Natl. Acad. Sci. USA.

[41] Liu Q., Lyubarsky A., Skalet J.H., Pugh E.N., Pierce E.A. (2003). RP1 is required for the correct stacking of outer segment discs. Invest. Ophthalmol. Vis. Sci..

