# Supplementary material for: Loss of the Metalloprotease ADAM9 Leads to Cone-Rod Dystrophy in Humans and Retinal Degeneration in Mice
Source: Am J Hum Genet. 2009 May 15;84(5):683–91. doi: 10.1016/j.ajhg.2009.04.005 (PMC2681008; doi:10.1016/j.ajhg.2009.04.005)
Supplement: Document S1. One Table and One Figure [file mmc1.pdf]

AJHG, Volume *84*

Supplemental Data

Loss of the Metalloprotease ADAM9 Leads to Cone-Rod Dystrophy in Humans and Retinal Degeneration in Mice

David A. Parry, Carmel Toomes, Lina Bida, Michael Danciger, Katherine V. Towns, Martin McKibbin, Samuel G. Jacobson, Clare V. Logan, Manir Ali, Jacquelyn Bond, Rebecca Chance, Steven Swendeman, Lauren L. Daniele, Kelly Springell, Matthew Adams, Colin A. Johnson, Adam P. Booth, Hussain Jafri, Yasmin Rashid, Eyal Banin, Tim M. Strom, Debora B. Farber, Dror Sharon, Carl P. Blobel, Edward N. Pugh Jr., Eric A. Pierce, and Chris F. Inglehearn

Table S1. Autozygosity mapping of SNPs and microsatellite markers in the CORD9 region.
SNPs and microsatellites were analyzed for homozygosity in 2 affected members of the Brazilian CORD9 family, indicated by arrows in Figure 2C. Where possible alleles are given, and where alleles differ between genotyped individuals the alleles from the left sibship are given first. The size of putative homozygous regions are shown as the distance between heterozygous markers. The 2.95 Mb refined CORD9 region is highlighted in green containing 82 homozygous markers, including 5 highly polymorphic microsatellites, at an average density of 1 every 0.034 Mb.

SNP/Microsatellite ID	Position (bp)	Genotype	Distance Between Heterozygous Markers (bp)	Alleles
**D8S1048**	26867404	Heterozygous	-	202/210 : 202/206
**D8S1820**	28053266	Heterozygous	1185862	113/117 : 113/105
**D8S1809**	28247294	Heterozygous	194028	161/161 : 161/172
rs924184	28352090	Heterozygous	104796	T/T : C/T
rs352226	28465958	Heterozygous	113868	A/G : A/A
rs893307	28560267	Homozygous		G/G
rs743673	28687510	Homozygous		C/C
rs1113990	28758718	Heterozygous	292760	C/G
rs2137473	28902480	Homozygous		C/C
rs6420164	29001999	Homozygous		C/C
rs1463363	29105837	Homozygous		T/T
rs559132	29200165	Heterozygous	441447	A/G
rs7341661	29250497	Homozygous		
rs2341674	29254175	Homozygous		C/C
rs4732927	29259703	Homozygous		T/T
rs4732952	29379234	Homozygous		T/T
rs10089171	29457959	Homozygous		A/A
rs7012771	29487626	Homozygous		G/G
rs4443701	29496754	Homozygous		G/G
rs6558129	29525525	Homozygous		C/C
rs4732980	29529350	Homozygous		G/G
rs4732986	29541846	Homozygous		C/C
rs6558134	29546527	Heterozygous	346362	C/T
rs9693716	29559782	Homozygous		
rs7845360	29561527	Homozygous		T/T
rs3935963	29572447	Homozygous		G/G
rs6558135	29577601	Homozygous		C/C
rs12546747	29583258	Homozygous		A/A
rs6558139	29586398	Homozygous		G/G

rs7002486	29597821	Heterozygous	51294	C/T
rs10095128	29602407	Homozygous		G/G
rs10089914	29680336	Homozygous		C/C
rs7459662	29802177	Homozygous		A/A
rs4464924	29806735	Homozygous		T/T
rs11994715	29807931	Homozygous		T/T
rs7832723	29917547	Homozygous		G/G
rs16916	29961444	Heterozygous	363623	A/T
rs10088363	30011411	Homozygous		C/C
rs7829853	30143082	Homozygous		G/G
**D8S1223**	30149650	Homozygous		127/127
rs3028786	30186216	Homozygous		
rs7001463	30286193	Homozygous		
rs4733476	30288664	Homozygous		T/T
rs4260861	30301880	Homozygous		
rs954003	30394679	Homozygous		T/T
rs4285443	30446859	Heterozygous	485415	A/G
**D8S339**	30476813	Homozygous		174/174
rs2979510	30509747	Heterozygous	62888	A/G
rs716942	30573674	Homozygous		
rs1116003	30774905	Homozygous		
rs2080719	30871664	Homozygous		
**D8S1021**	30923457	Heterozygous	413710	ab
rs7006039	30935824	Homozygous		
rs1362911	30984486	Homozygous		
rs1477535	31078407	Heterozygous	154950	A/G
rs2019726	31087966	Homozygous		
rs2252480	31109652	Heterozygous	31245	C/T
rs3024239	31118664	Heterozygous	9012	C/T
rs6468026	31194597	Homozygous		C/C
rs10447952	31216808	Homozygous		A/A
rs7812391	31241311	Heterozygous	122647	C/T
rs4609156	31253200	Homozygous		T/T
**D8S1770**	31259325	Homozygous		260/260
rs4733071	31260412	Homozygous		
**D8S1769**	31264876	Homozygous		246/246
rs5002658	31270556	Homozygous		
rs1992061	31295271	Homozygous		
rs7460665	31316591	Homozygous		
rs2681603	31323014	Homozygous		T/T
rs10093871	31338282	Homozygous		
rs6468041	31354330	Homozygous		G/G
rs737377	31368530	Homozygous		T/T
rs2575069	31374239	Homozygous		G/G

rs6996092	31427599	Homozygous		
rs2033671	31440415	Homozygous		G/G
**D8S1711**	31443817	Homozygous		141/141
rs7828595	31452978	Heterozygous	211667	C/T
rs7844630	31458798	Heterozygous	5820	A/G
rs7834980	31463987	Homozygous		A/A
rs2681627	31469010	Homozygous		T/T
rs763551	31564671	Heterozygous	105873	A/C
rs7002293	31721966	Homozygous		T/T
rs10109763	31791935	Homozygous		T/T
**D8S1810**	31811263	Homozygous		185/185
rs10503892	31824975	Homozygous		T/T
rs7357490	31870435	Homozygous		C/C
**D8S2319**	31934883	Heterozygous	370212	130/138
**D8S1477**	32186709	Heterozygous	251826	159/167
**D8S499**	32261497	Heterozygous	74788	149/145
D8S1125	32487058	Homozygous		232/232
**D8S375**	32577362	Heterozygous	315865	ba
**D8S1758**	33172329	Heterozygous	594967	236/250
**D8S283**	33747507	Heterozygous	575178	115/119
**D8S513**	33788903	Heterozygous	41396	202/198
**D8S505**	34570479	Heterozygous	781576	203/209
**D8S1750**	35532837	Heterozygous	962358	212/219
**D8S1121**	35920931	Heterozygous	388094	114/117
**D8S1700**	35921521	Heterozygous	590	120/122
**D8S1803**	36514271	Heterozygous	592750	280/284
**D8S1747**	37112470	Heterozygous	598199	218/220
rs502356	37168224	Heterozygous	55754	A/G
rs374241	37273182	Homozygous		
rs239429	37353994	Heterozygous	185770	A/G
rs2541434	37382893	Heterozygous	28899	A/G
rs2843797	37388099	Heterozygous	5206	C/T
rs2843781	37400574	Heterozygous	12475	C/T
rs2541454	37405319	Homozygous		T/T
rs7812562	37419329	Heterozygous	18755	C/T
rs7017160	37448473	Heterozygous	29144	G/T
rs16886775	37450213	Heterozygous	1740	A/C
rs16886778	37450348	Heterozygous	135	C/T
rs7001685	37452990	Heterozygous	2642	C/T
rs9642773	37463440	Heterozygous	10450	C/T
rs10955025	37473135	Heterozygous	9695	A/G
rs12543429	37550490	Homozygous		C/C
rs7832424	37612663	Homozygous		A/A
rs7814863	37690550	Homozygous		
rs7005432	37767164	Homozygous		T/T
rs915650	37776684	Homozygous		C/C
rs4976893	37780106	Homozygous		C/C

**D8S1722**	37780634	Homozygous		217/217
rs7813990	37790298	Homozygous		T/T
rs6998793	37816032	Homozygous		T/T
rs12541064	37825425	Homozygous		A/A
rs7341564	37849614	Homozygous		T/T
rs10955035	37857774	Homozygous		G/G
rs7016233	37858061	Homozygous		A/A
rs7812866	37869447	Homozygous		C/C
rs7833285	38119476	Homozygous		
rs2843745	38143780	Homozygous		T/T
rs12682626	38165926	Homozygous		A/A
rs7836805	38182392	Homozygous		C/C
**D8S1791**	38273218	Homozygous		239/239
rs7835269	38346917	Homozygous		
rs6987534	38418872	Homozygous		G/G
**D8S1821**	38470657	Homozygous		150/150
rs7845393	38478625	Homozygous		C/C
rs1541897	38482629	Homozygous		A/A
rs2461327	38493081	Homozygous		T/T
rs2445026	38528479	Homozygous		T/T
rs7817828	38549391	Homozygous		G/G
rs1000236	38593641	Homozygous		A/A
**GATA101H09**	38759531	Homozygous		287/287
rs6997835	38776852	Homozygous		T/T
rs7818605	38783331	Homozygous		A/A
rs2037205	38803584	Homozygous		A/A
rs7005388	38806387	Homozygous		C/C
rs869471	38813540	Homozygous		C/C
rs7009521	38820486	Homozygous		G/G
rs4577922	38828559	Homozygous		
rs3847141	38873687	Homozygous		A/A
rs4301415	38943408	Homozygous		
rs10809000	39006349	Homozygous		G/G
rs7818363	39017964	Homozygous		C/C
rs7015505	39021034	Homozygous		T/T
rs4075849	39053740	Homozygous		C/C
rs6474525	39056144	Homozygous		T/T
rs7840009	39131609	Homozygous		A/A
rs10094307	39188310	Homozygous		T/T
rs4551318	39261707	Homozygous		
rs7006167	39302883	Homozygous		G/G
rs6474131	39344125	Homozygous		C/C
rs6474159	39577556	Homozygous		

rs11985352	39586481	Homozygous		A/A
rs6983284	39642225	Homozygous		G/G
rs10103827	39647959	Homozygous		A/A
rs6474166	39659073	Homozygous		G/G
rs1901388	39672418	Homozygous		T/T
rs9657175	39692225	Homozygous		A/A
rs6474169	39697120	Homozygous		T/T
rs10087294	39738317	Homozygous		C/C
rs7835497	39741907	Homozygous		T/T
rs3853758	39744837	Homozygous		G/G
rs2290301	39753124	Homozygous		T/T
rs13253873	39758568	Homozygous		G/G
rs7822312	39764090	Homozygous		C/C
rs2100309	39766809	Homozygous		A/A
rs7812966	39769578	Homozygous		
rs1451741	39770097	Homozygous		C/C
rs7821582	39781596	Homozygous		C/C
rs6474177	39783853	Homozygous		
rs7832788	39837739	Homozygous		A/A
rs3808606	39888532	Homozygous		T/T
rs7820268	39896686	Homozygous		C/C
rs3739319	39904478	Homozygous		A/A
rs6992375	39909565	Homozygous		C/C
rs11777027	39916628	Homozygous		C/C
rs4736969	39984685	Homozygous		A/A
**D8S255**	40004275	Homozygous		117/117
rs1383603	40043175	Homozygous		A/A
rs2955862	40063231	Homozygous		G/G
rs10199	40131220	Homozygous		G/G
rs6474228	40143763	Homozygous		G/G
rs724323	40158021	Homozygous		A/A
rs726922	40204591	Homozygous		A/A
rs6984370	40327248	Homozygous		G/G
rs725401	40420045	Heterozygous	2946910	C/T
**D8S1817**	40567202	Heterozygous	147157	176/168
**D8S1104**	40762949	Heterozygous	195747	133/137
**D8S532**	40907529	Heterozygous	144580	243/245
**D8S589**	51088398	Heterozygous	10180869	145/141

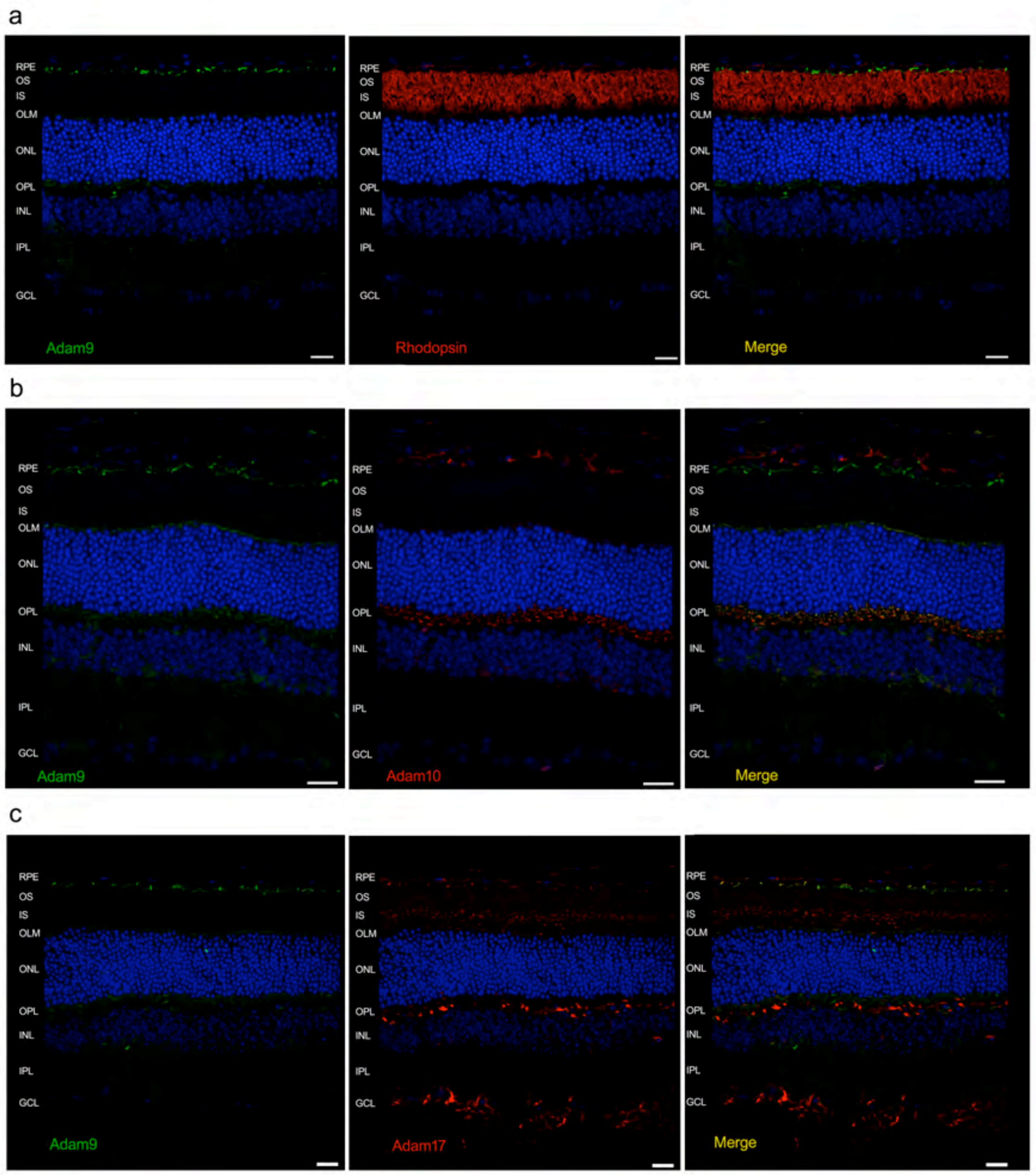

Figure S1. Adam9 staining of mouse retina.
Mouse eye cryosections were prepared by standard methods and incubated with a goat polyclonal antibody to Adam9 (Santa Cruz Biotechnology inc., SC23290) and co-stained with antibodies to **(a)** Rhodopsin (Sigma, R9153), **(b)** Adam10 (Chemicon International, AB 19026) or **(c)** Adam17 (R & D Systems, MAB9302). Bound Adam9 primary antibody was detected with Alexa Fluor 488 conjugated secondary antibody. Rhodopsin, Adam10 and Adam17 antibodies were detected with Alexa Fluor 594 conjugated secondary antibody. DAPI staining of nuclei is shown in blue. Adam9 immunoreactivity shows brightest staining at the RPE - POS boundary, but cross-reactivity was observed in some *Adam9*$^{-/-}$ retinal sections (not shown) so these data should be interpreted with caution. Adam9,

Adam10 and Adam17 show distinct staining patterns but some Adam17 staining is also observed at the RPE – POS interface. Adam9 immunoreactivity in the *Adam9*$^{-/-}$ retina could be due to cross-reactivity of the antibody with other Adam family members at this location. RPE, retinal pigment epithelium; OS, photoreceptor outer segments; IS, photoreceptor inner segments; OLM, outer limiting membrane; ONL, outer nuclear layer; OPL, outer plexiform layer; INL, inner nuclear layer; IPL, inner plexiform layer; GCL, ganglion cell layer. Scale bars: 20 μm.
